# Donor MHC-specific thymus vaccination allows for immunocompatible allotransplantation

**DOI:** 10.1038/s41422-024-01049-5

**Published:** 2025-01-03

**Authors:** Yang Liu, Hexi Feng, Ke Li, Ruiyi Li, Xiao-Jie Zhang, Ye Tian, Yujiang Fang, Yanjie Zhou, Ling Liu, Xiaoqing Zhang

**Affiliations:** 1 Translational Medical Center for Stem Cell Therapy, Shanghai East Hospital, School of Medicine, Tongji University, Shanghai, China; 2 Translational Research Institute of Brain and Brain-Like Intelligence, Shanghai Fourth People’s Hospital, School of Medicine, Tongji University, Shanghai, China; 3 Shanghai Institute of Stem Cell Research and Clinical Translation, Shanghai, China; 4 Stem Cell Research Center and Key Laboratory of Neuroregeneration of Shanghai Universities, School of Medicine, Tongji University, Shanghai, China; 5 Clinical Center for Brain and Spinal Cord Research, Tongji University, Shanghai, China; 6 Department of Gynaecology, Jing’an District Hospital of Traditional Chinese Medicine, Shanghai, China; 7 School of Foreign Studies, Tongji University, Shanghai, China; 8 Key Laboratory of Spine and Spinal Cord Injury Repair and Regeneration of Ministry of Education, Orthopaedic Department of Tongji Hospital, Shanghai, China

**Keywords:** Immunology, Embryonic stem cells

## Abstract

Organ transplantation is the last-resort option to treat organ failure. However, less than 10% of patients benefit from this only option due to lack of major histocompatibility complex (MHC)-matched donor organs and 25%–80% of donated organs could not find MHC-matched recipients. T cell allorecognition is the principal mechanism for allogeneic graft rejection. We herein present a “donor MHC-specific thymus vaccination” (DMTV) strategy to induce T cell tolerance to both autologous and allogeneic donor MHC. Allogeneic MHC molecules were expressed in the recipient thymus through adeno-associated virus-mediated delivery, which led to stable expression of allogeneic MHC together with the autologous MHC in the engineered thymus. During local T cell education, those T cells recognizing either autologous MHC or allogeneic MHC were equally depleted. We constructed C57BL/6-MHC and BALB/c-MHC dual immunocompatible mice via thymus vaccination of C57BL/6-MHC into the BALB/c thymus and observed long-term graft tolerance after transplantation of C57BL/6 skin and C57BL/6 mouse embryonic stem cells into the vaccinated BALB/c mice. We also validated our DMTV strategy in a bone marrow, liver, thymus (BLT)-humanized mouse model for immunocompatible allotransplantation of human embryonic stem cells. Our study suggests that the DMTV strategy is a potent avenue to introduce a donor compatible immune system in recipients, which overcomes the clinical dilemma of the extreme shortage of MHC-matched donor organs for treating patients with end-stage organ failure.

## Introduction

Organ transplantation is the last-resort option to treat patients with end-stage organ failure. According to 2021 global report published by the Global Observatory on Donation and Transplantation,^1^ less than 10% of patients left with this only option received organ transplantation treatment owing to an extreme shortage of major histocompatibility complex (MHC, human MHC is generally recognized as human leukocyte antigens or HLA)-matched donor organs. On the other hand, the acceptance rate is just 0.6% in U.S. when the electronically transmitted offers for patients are ahead of the ultimate acceptor, and the nonuse rate among recovered organs for transplant or organs potentially available per donor approaches 25%–80%.^2–4^ This organ underutilization is attributable to the post-donation challenges to the allocation, storage and transport of recovered organs to waitlisted and MHC-matched patients.^5,6^

MHC mismatch is the leading cause of transplant rejection.^5,7–10^ There are two major types of MHC molecules. MHC class I (MHC I) molecules are expressed on the surface of all nucleated cells while MHC class II (MHC II) molecules are restricted to professional antigen-presenting cells (APCs).^11,12^ Allogeneic MHC molecules expressed on donor cells are directly recognized by T cell receptors (TCRs) of recipient T cells after organ transplantation, which is the primary mechanism for immune rejection.^13–17^ MHC I and MHC II also present antigen peptides periodically broken down during normal or diseased cellular processes to the immune cells for antigen recognition, which partially contributes to immune rejection.^18,19^ Successful organ transplantation largely relies on the degree of MHC matching.^5,10^ However, as MHC is highly polymorphic, fully MHC-matched donors are rarely available.^20,21^ Therefore, immunosuppressants are regularly used to reduce the intensity of immune responses after allotransplantation, although this might lead to increased risk of infections and malignancies.^22–24^

T cells are central to the process of transplant rejection. The features of conventional T cells discriminating between MHC-mismatched “non-self” cells and MHC-matched “self” cells are acquired during their thymic development.^25–27^ In the thymus, immature T cells (thymocytes) undergo a positive-negative selection.^27–30^ During positive selection, T cells undergo apoptosis by neglect when there is a low affinity between their surface TCRs and MHCs expressed on thymic epithelial cells (TECs) and dendritic cells (DCs).^31–35^ Those T cells are also programmed to undergo apoptosis during negative selection when TCRs on immature T cells bind with a high affinity to MHC or presented antigenic peptides in order to prevent autoimmune response.^31,36–38^

Here, we report a donor MHC-specific thymus vaccination (DMTV) strategy to recapitulate TCR-MHC adaptation during T cell development in the thymus. We hypothesize that ectopic expression of allogeneic MHC (MHC^allo^) in TECs and DCs in recipient thymus will drive “non-self” to “self” T cell antigen discrimination via specific depletion of the donor-reactive T cells during positive and negative selection. The recipient receiving DMTV is expected to tolerate allotransplantation of the donor organs or tissues bearing the allelic variants of vaccinated MHC with no immunosuppressant required.

## Results

### Ectopic expression of allogeneic MHC in recipient thymus

**Fig. 1:**
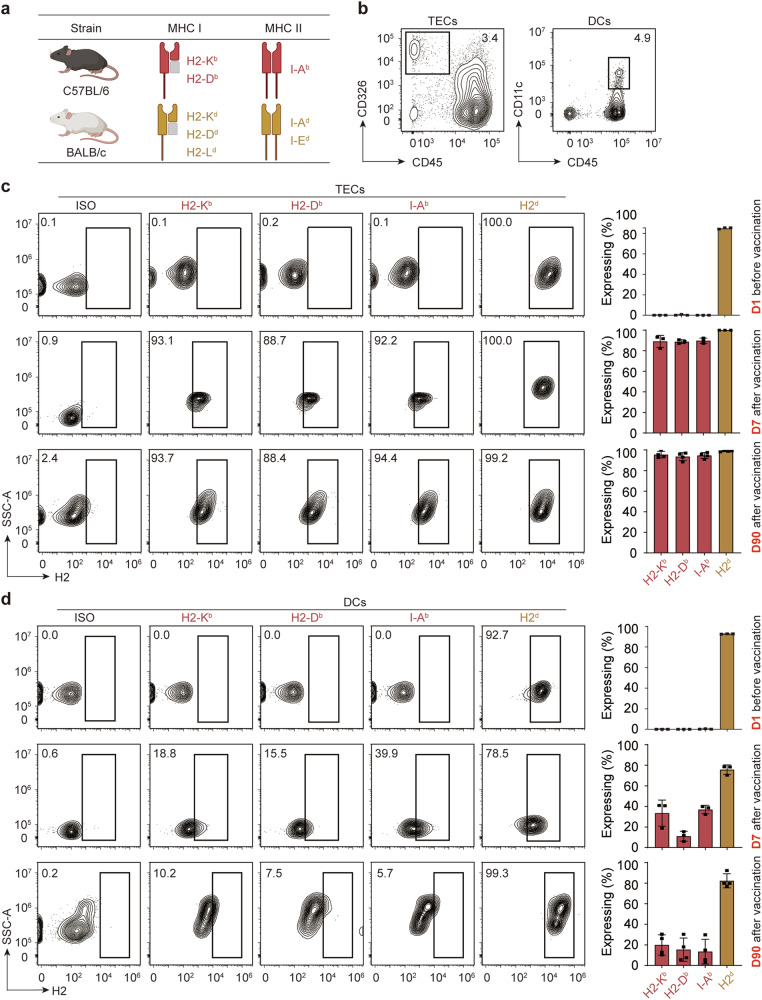
Efficient expression of allogeneic MHC in TECs and DCs for DMTV. **a** MHC profiles of C57BL/6 mice and BALB/c mice. **b** FACS analysis of TECs and DCs in the thymus of BALB/c mice after DMTV of C57BL/6 mice MHC (DMTV^C57^). **c** Representative flow cytometry plots illustrating TECs expressing self-MHC (BALB/c) and donor-MHC (C57BL/6) at day 1 before vaccination and day 7 or day 90 after vaccination (left panel), and percentages of cells expressing self-MHC and donor-MHC are quantified (right panel). Data are mean ± SEM (*n* = 3 independent experiments). **d** Representative flow cytometry plots illustrating DCs expressing self-MHC and donor-MHC at day 1 before vaccination and day 7 or day 90 after vaccination (left panel), and percentages of cells expressing self-MHC and donor-MHC are quantified (right panel). Data are mean ± SEM (*n* = 3 independent experiments).

BALB/c (recipient) and C57BL/6 (donor) mice are two MHC completely mismatched allogeneic strains, and C57BL/6 mice of the H2^b^ haplotype do not express the MHC I molecule H2-L or the MHC II molecule I-E^39^ (Fig. 1a). AAV2/8-CMV-H2-K^b^ α chain-IRES-H2-D^b^ α chain and AAV2/8-CMV-I-A^b^ α chain-IRES-I-A^b^ β chain bearing both C57BL/6 donor MHC I and MHC II expression cassettes were constructed and viruses were then packaged and concentrated for thymus vaccination (Supplementary information, Fig. S1). 1 × 10^11^ viral genomes (vg) for each virus in 10 μL were intrathymically injected for each lobe of BALB/c mouse (DMTV^C57^), and an empty AAV2/8 virus was similarly injected in the thymus at a concentration of 2 × 10^11^ vg for each lobe for control (Ctrl TV). 7 days after injection, C57BL/6 donor MHC I H2-K^b^ and H2-D^b^ as well as MHC II I-A^b^ were detected in 88.7%–93.1% TECs and 15.5%–39.9% DCs in the vaccinated BALB/c thymus as evaluated by fluorescence-activated cell sorting (FACS) (Fig. 1b–d). The ectopic expression of donor MHC in TECs lasted for more than 90 days, although those expressed in DCs eventually decreased, which is likely due to the rapid turnover rate of DCs in the thymus (Fig. 1c, d).^40^ These data suggest that the delivery system was efficient and the stable expression pattern of donor MHC in TECs of recipients would therefore ensure a long-term effect for introducing donor MHC tolerance.

### DMTV leads to alloreactive T cell clonal depletion

**Fig. 2:**
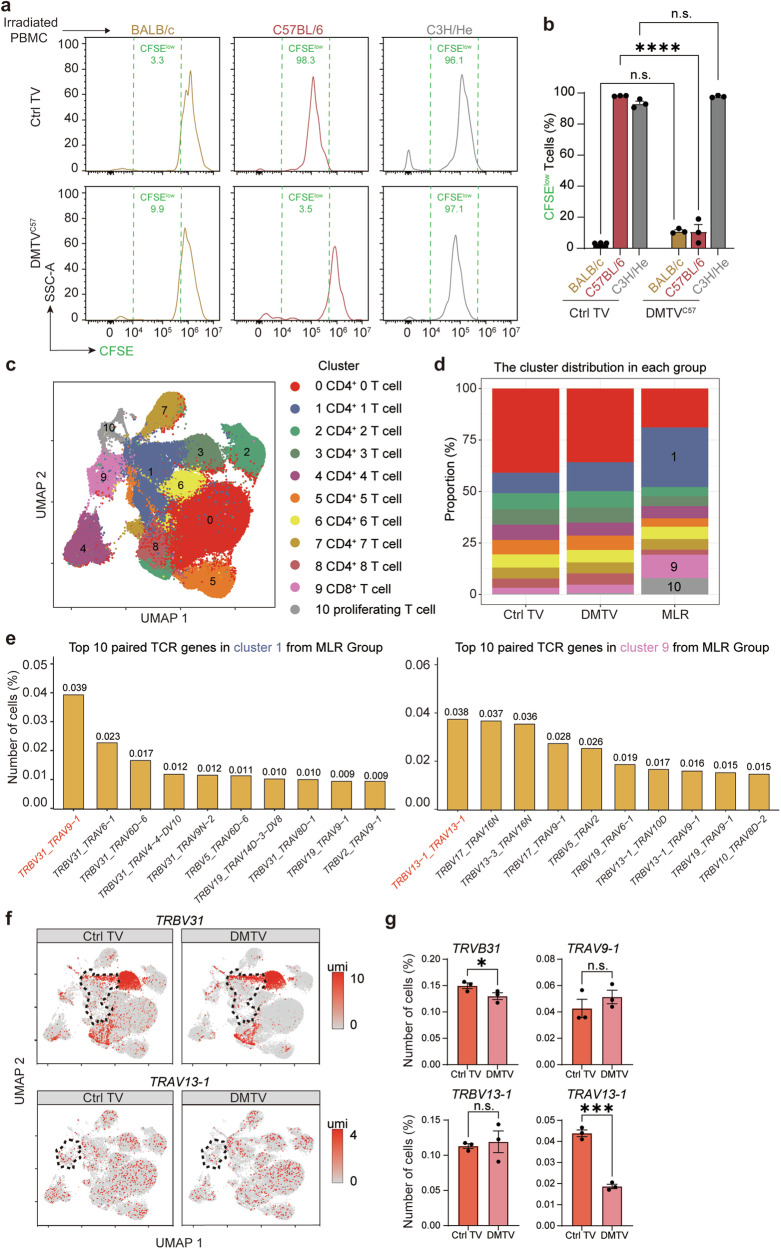
Clonal depletion of donor-reactive T cells mediated by DMTV. **a** PBMCs from Ctrl TV BALB/c mice or DMTV^C57^ BALB/c mice were labeled with CFSE and primed with irradiated PBMCs from BALB/c mice, C57BL/6 mice or C3H/He mice. CD3^+^CFSE^low^ T cells were then analyzed by FACS. **b** Quantification of proportions of CD3^+^CFSE^low^ clonally expanded T cells following allogeneic MHC stimulation. Data are mean ± SEM (*n* = 3 independent experiments). Statistical significance was determined using the one-way ANOVA followed by Dunnett’s comparisons test. ****P < 0.0001; n.s., non-significant. **c** Clonally expanded T cells in mixed lymphocyte reaction (MLR, *n* = 2, 23,709 cells) and peripheral T cells from Ctrl TV (*n* = 3, 383,485 cells) and DMTV^C57^ BALB/c mice (*n* = 3, 330,241 cells) were subjected to integrated scRNA-seq & scTCR-seq, and were categorized into 11 clusters as visualized by UMAP plot. **d** Quantification of proportions of each cluster in **c**. Clusters #1, #9 and #10 were clonally expanded in MLR after priming with irradiated C57BL/6 PBMCs. **e** TRAV-TRBV pairing profiles in clonally expanded Cluster #1 T cells (left panel) and Cluster #9 T cells (right panel) in MLR. **f** FeaturePlot analyses showed representative *TRBV31* and *TRAV13-1* TCR gene expression in T cell clusters from Ctrl TV and DMTV^C57^ BALB/c mice. Downsampling to 42,130 cells in total and 3991 cells in Cluster #1 in Ctrl TV group, and 30,241 cells in total and 4082 cells in Cluster #1 in the DMTV^C57^ group (top panel); downsampling to 35,755 cells in total and 1016 cells in Cluster #9 in Ctrl TV group, and 30,241 cells in total and 1054 cells in Cluster #9 in the DMTV^C57^ group (lower panel). Clusters #1 and # 9 of interest were broken line circled. **g** Quantification of proportions of *TRBV31* and *TRAV9-1* TCR-bearing T cells in Cluster #1, and *TRAV13-1* and *TRBV13-1* TCR-bearing T cells in Cluster #9 in Ctrl TV and DMTV^C57^ groups. Data are mean ± SEM (*n* = 3 independent experiments). Statistical significance was determined using two-tailed unpaired Student’s *t*-test. ***P < 0.001; *P < 0.05; n.s., non-significant.

To evaluate the functional outcome of DMTV, BALB/c mice that received Ctrl TV or DMTV^C57^ for 7 days were treated with anti-CD4 and anti-CD8 monoclonal antibodies (mAbs) as well as 3 Gy total body irradiation (TBI) to eliminate T cell repertoire, including pre-existing donor-reactive T cell populations. Newly developed CD4^+^ T cells and CD8^+^ T cells completely reconstituted the cell repertoire in peripheral 2 months after depletion (Supplementary information, Fig. S2). The reconstituted peripheral blood mononuclear cells (PBMCs) from Ctrl TV and DMTV^C57^ BALB/c mice were then used for mixed lymphocyte reaction (MLR) analysis to evaluate their reactiveness to donor cells (Supplementary information, Fig. S3a). On the one hand, PBMCs from both Ctrl TV and DMTV^C57^ BALB/c mice did not show any responsiveness to irradiated PBMCs from BALB/c mice (Fig. 2a, b; Supplementary information, Fig. S3b), suggesting that no autoimmunity is acquired after the process of thymus vaccination of either Ctrl or allogeneic MHC. We also detected serum anti-nuclear antibodies (ANAs) in DMTV^C57^ BALB/c mice. Results showed that ANAs in DMTV mice were comparable with those in the normal saline thymus injected and Ctrl TV mice (Supplementary information, Fig. S3c), which further excluded the possibility of autoimmune responses potentially induced by DMTV. On the other hand, PBMCs from both groups equally responded to common antigen phytohemagglutinin (PHA) and irradiated PBMCs from C3H/He mice, another mouse strain with distinct MHC background (H2^k^) (Fig. 2a, b; Supplementary information, Fig. S3b), suggesting normal immune responsiveness remained in both Ctrl and DMTV mice. Remarkably, PBMCs from DMTV^C57^ BALB/c mice had a much lower responsiveness towards irradiated PBMCs from C57BL/6 mice as compared with that of PBMCs from Ctrl TV BALB/c mice (Fig. 2a, b). These data strongly suggest that DMTV results in efficient and specific T cell tolerance towards cells expressing vaccinated MHC variants.

To confirm specific depletion of donor-reactive T cells, clonally expanded T cells from PBMCs of Ctrl TV BALB/c mice were FACS enriched and subjected to integrative single-cell RNA sequencing and TCR sequencing (scRNA-seq & scTCR-seq) after priming with irradiated C57BL/6 PBMCs (Fig. 2a; Supplementary information, Fig. S3a, d). As compared with the non-primed T cells, a cluster of CD4^+^ T cells (cluster #1) and CD8^+^ T cells (cluster #9) were specifically expanded after priming with irradiated C57BL/6 PBMCs (Fig. 2c, d; Supplementary information, Fig. S3e). Meanwhile, proliferating T cells (cluster #10) were also obviously elevated after priming with allogeneic PBMCs (Fig. 2d; Supplementary information, Fig. S3e). The most dominant TCR profiles in clonally expanded CD4^+^ T cells and CD8^+^ T cells were then successfully retrieved and considered as the potential candidates that specifically responded to donor MHC (Fig. 2e; Supplementary information, Fig. S3f). We then isolated peripheral T cells from Ctrl TV BALB/c mice and DMTV^C57^ BALB/c mice 3 months after vaccination for integrated scRNA-seq & scTCR-seq. There were no differences on the overall constitutions in peripherally re-populated T cells after thymus vaccination (Fig. 2d),^36^ suggesting that intact intrinsic T cell developmental programs remained after DMTV. Intriguingly, the proportions of *TRBV31*-bearing CD4^+^ T cells in cluster #1 and *TRAV13-1*-bearing CD8^+^ T cells in cluster #9 were significantly reduced in DMTV mice (Fig. 2f, g). Together, these results indicate that donor-reactive T cells are specifically depleted during T cell development in the thymus of mice receiving DMTV.

### DMTV leads to blunted immune responses to donor cells in vivo

**Fig. 3:**
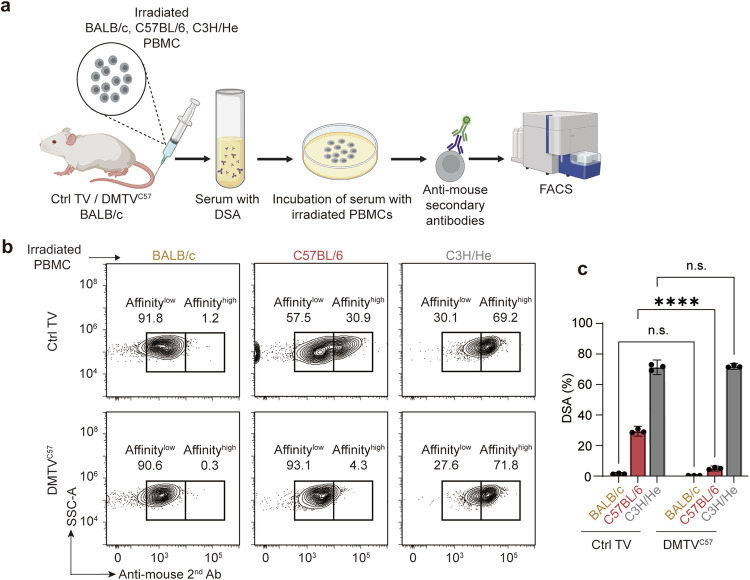
Donor-specific tolerance mediated by DMTV. **a** Schematic representation of designed processes for detecting DSAs. Irradiated PBMCs from BALB/c, C57BL/6 or C3H/He mice were tail vein injected into Ctrl TV and DMTV^C57^ BALB/c mice. 10 days after inoculation, DSAs in the serum were monitored by incubation with irradiated PBMCs isolated from BALB/c, C57BL/6, or C3H/He mice, incubation with FITC-conjugated anti-mouse secondary antibody and FACS analysis. **b** Representative flow cytometry plot of DSA detection, where Affinity^high^ indicates the presence of DSAs in the serum. **c** Quantification of proportions of cells with high DSA affinity in **b**. Data are mean ± SEM (*n* = 3 independent experiments). Statistical significance was determined using the one-way ANOVA followed by Dunnett’s comparisons test. ****P < 0.0001; n.s., non-significant.

After allotransplantation, T cells are initially activated either directly or indirectly, which subsequently leads to activation of B cells, resulting in both cell-mediated and antibody-mediated immune rejection.^41^ Donor-specific antibody (DSA) monitoring is therefore widely used for detecting the overall levels of immune rejection and a guide for tailored immunosuppressive treatment.^42,43^ To analyze the immune responses to donor cells after DMTV in vivo, we monitored DSAs in Ctrl TV and DMTV^C57^ BALB/c mice (Fig. 3a). After tail vein injection of irradiated PBMCs from BALB/c mice for 10 days, no DSA was detected in the sera of both Ctrl TV and DMTV^C57^ BALB/c mice (Fig. 3b, c). As expected, the injection of irradiated PBMCs from C3H/He mice resulted in comparable and prominent induction of DSA in both groups. On the other hand, the injection of irradiated C57BL/6 PBMCs led to robust DSA induction in Ctrl TV BALB/c mice and DMTV^C57^ treatment almost fully abolished DSA induction (30% vs 5% of incubated PBMCs labeled with DSA for DMTV^C57^ group vs Ctrl TV group) (Fig. 3b, c), highlighting the robustness of DMTV in blunting the overall donor-specific immune responses in vivo.

### DMTV mitigates immune rejection of skin allotransplants

**Fig. 4:**
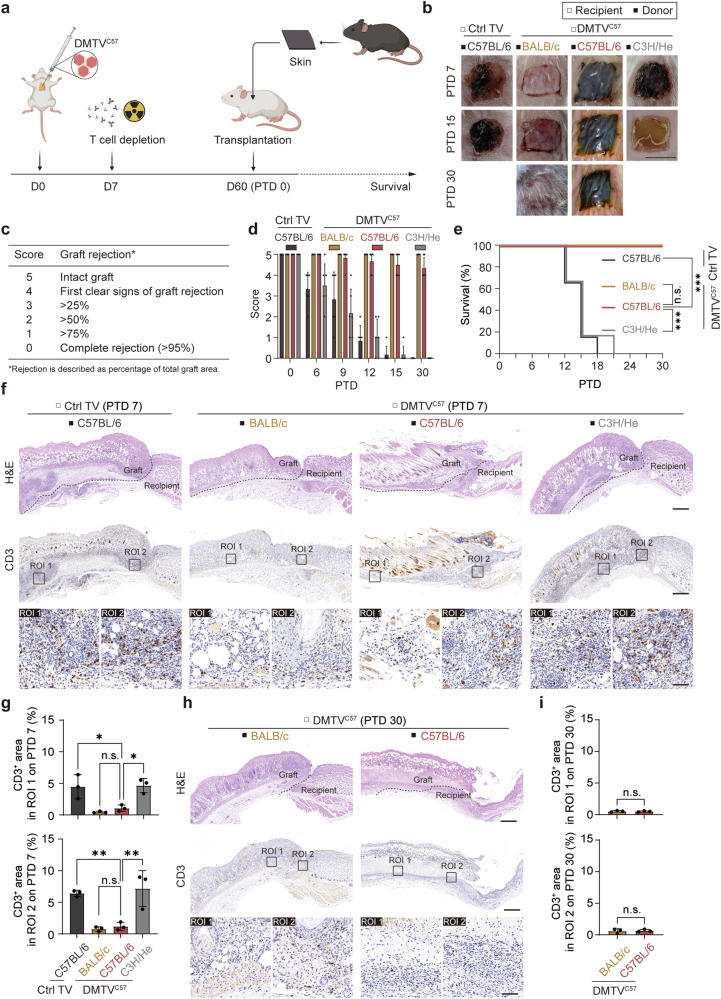
Donor-specific tolerance of allotransplanted skin mediated by DMTV. **a** Schematic representation of skin transplantation in thymus vaccinated mice. Day 0, thymus vaccination of Ctrl or C57BL/6 MHC; day 7, T cell depletion via anti-T cell antibodies and irradiation; day 60 (post-transplantation day 0, PTD 0), skin transplantation. Recipient, BALB/c mice; donor, BALB/c mice, C57BL/6 mice or C3H/He mice. **b** Representative skin tissues 7, 15 or 30 days after transplantation. 30 days after transplantation, no grafts survived in Ctrl TV BALB/c mice transplanted with C57BL/6 skin or in DMTV^C57^ BALB/c mice transplanted with C3H/He skin. Scale bar, 1 cm. **c** 5-point rating scale for evaluation of the status of the donor skin grafts. **d** Survival scores of the donor skin grafts. Data are mean ± SEM (*n* = 6 independent experiments). **e** Survival curves of the donor skin grafts. Data are mean ± SEM (*n* = 6 independent experiments). ***P < 0.001; n.s., non-significant; Log-rank test. **f** Representative H&E and IHC staining of CD3^+^ T cells in adjacent slices from mice 7 days post-transplantation. Region of interest 1 (ROI 1), the region of the donor skin graft; ROI 2, the junction region between the recipient skin and the donor skin. Scale bars of the top two panels, 500 μm; scale bar of the lower panel, 40 μm. **g** Quantification analyses of CD3-positively stained area 7 days after transplantation in ROI 1 and ROI 2 in **f**. Data are mean ± SEM (*n* = 3 independent experiments). Statistical significance was determined using the one-way ANOVA followed by Dunnett’s comparisons test. **P < 0.01; *P < 0.05; n.s., non-significant. **h** Representative H&E and IHC staining of CD3^+^ T cells in adjacent slices from mice 30 days after transplantation. ROI 1, the region of the donor skin graft; ROI 2, the junction region between the recipient skin and the donor skin. Scale bars of the top two panels, 500 μm; scale bar of the lower panel, 40 μm. **i** Quantification analyses of CD3-positively stained area 30 days after transplantation in ROI 1 and ROI 2 in **h**. Data are mean ± SEM (*n* = 3 independent experiments). Statistical significance was determined using two-tailed unpaired Student’s *t*-test. n.s., non-significant.

To examine whether DMTV could provide an immune competent while donor-specific immunotolerant condition for allotransplantation, we transplanted the skin from C57BL/6 mice to BALB/c mice 2 months after thymus vaccination (Fig. 4a). In Ctrl TV BALB/c mice, the transplanted skin from C57BL/6 mice showed prominent rejection starting from post transplantation day (PTD) 6 (Fig. 4b–e). The transplanted skin from C3H/He mice was similarly rejected in DMTV^C57^ BALB/c mice on PTD 6–15. Comparable to that in autologous transplantation, the transplanted skin donated from C57BL/6 mice in DMTV^C57^ BALB/c mice showed tolerance 30 days after transplantation (Fig. 4b–e). Even 100 days after transplantation, we could still observe the donor C57BL/6 skin tissues at the graft site (Supplementary information, Fig. S4a). These data suggest that DMTV constructs a donor-specific immune tolerance environment and supports long-term survival of allotransplanted organs with no need of immunosuppressive treatment.

Histological analyses were performed to check immune cell infiltration after transplantation. There were massive CD3^+^ T cells, CD4^+^ T cells and CD8^+^ T cells infiltrated in the C57BL/6 mice skin tissues transplanted into the Ctrl TV BALB/c mice and the C3H/He mice skin tissues transplanted into DMTV^C57^ BALB/c mice (Fig. 4f–i; Supplementary information, Fig. S4b, c). Similar to the autologous transplantation group, very rare T cell infiltration was observed in the C57BL/6 mice skin tissues which were transplanted into the DMTV^C57^ BALB/c mice on PTD 7 or 30 (Fig. 4f–i; Supplementary information, Fig. S4b, c). The sera of DMTV^C57^ BALB/c mice 30, 60, and 90 days after C57BL/6 mouse skin tissue transplantation were also subjected to DSA detection, and results showed that less than 2% of incubated PBMCs were labeled with DSA at all three time points observed (Supplementary information, Fig. S4d, e), suggesting that donor-specific immune responses were largely and steadily blunted after DMTV treatment.

In clinical practice, before organ transplantation, patients may have been sensitized due to exposures to alloantigens, such as blood transfusion, pregnancies and previous transplantation.^44^ To verify whether the DMTV strategy can be applied to sensitized recipients, we transplanted skin tissues from C57BL/6 mice to naïve BALB/c mice, which led to expected graft rejection in 15 days. The sensitized BALB/c mice were then subjected to DMTV^C57^ followed by a secondary transplantation (Supplementary information, Fig. S5a). Again, the secondarily grafted skin tissues were well tolerated in thymus vaccinated BALB/c mice for more than 100 days with almost no DSA production (Supplementary information, Fig. S5b–g), suggesting the robustness of the DMTV strategy in applying in organ allotransplantation in recipients with complexed alloantigen exposure.

**Fig. 5:**
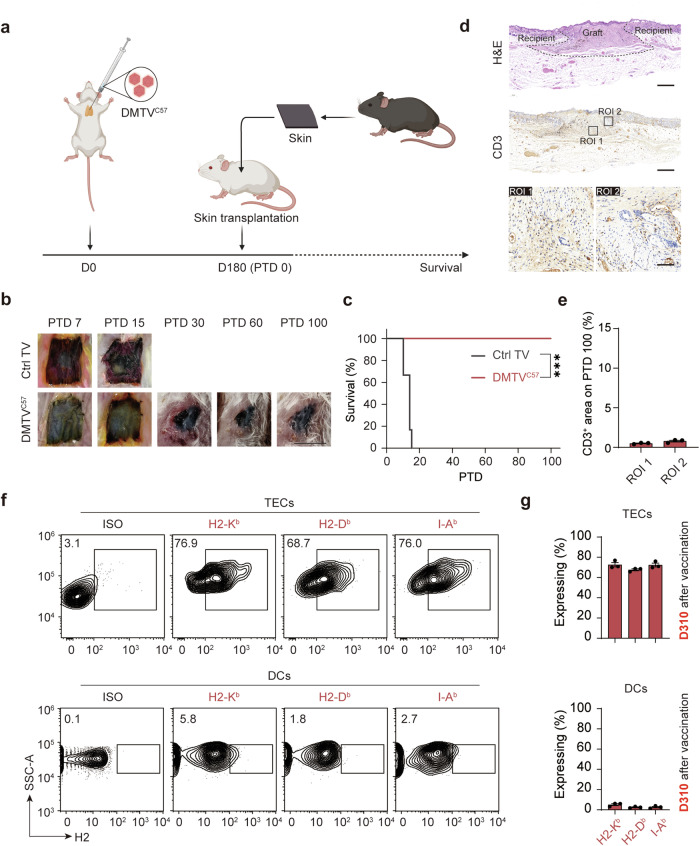
T cell depletion and TBI are not required for DMTV-induced tolerance. **a** Schematic representation of skin transplantation in thymus vaccinated mice without T cell depletion and TBI in advance. Day 0, thymus vaccination of Ctrl or C57BL/6 MHC; day 180 (PTD 0), skin transplantation. Recipient, BALB/c mice; donor, C57BL/6 mice. **b** Representative skin tissues 7, 15, 30, 60 or 100 days after transplantation. 30 days after transplantation, no grafts survived in Ctrl TV BALB/c mice transplanted with C57BL/6 skin. Scale bar, 1 cm. **c** Survival curves of the donor skin grafts. Data are mean ± SEM (*n* = 6 independent experiments). ***P < 0.001; Log-rank test. **d** Representative H&E and IHC staining of CD3^+^ T cells in adjacent slices from mice 100 days after transplantation. ROI 1, the region of the donor skin graft; ROI 2, the junction region between the recipient skin and the donor skin. Scale bars of the top two panels, 500 μm; scale bar of the lower panel, 40 μm. **e** Quantification analyses of CD3-positively stained area 100 days after transplantation in ROI 1 and ROI 2 in **d**. Data are mean ± SEM (*n* = 3 independent experiments). **f** Representative flow cytometry plots illustrating TECs and DCs expressing donor-MHC (C57BL/6) on day 310 after vaccination. **g** Percentages of cells expressing donor-MHC are quantified. Data are mean ± SEM (*n* = 3 independent experiments).

Selective depletion of T cells via anti-T cell antibody injection has always been used to reduce immune rejection in organ allotransplantation, while the elimination of the T cell repertoire is inevitably a concern. To study the effectiveness of the DMTV strategy in organ allotransplantation in recipients without T cell depletion or TBI in advance, we directly treated BALB/c mice with DMTV^C57^ and waited for 6 months, a time window sufficient for T cell turnover (Fig. 5a).^45^ It is worth noting that again skin tissues from C57BL/6 mice transplanted to DMTV^C57^-treated BALB/c mice showed long-term tolerance without DSA production (Fig. 5b–e; Supplementary information, Fig. S5h, i). Meanwhile, ectopic donor MHC molecules were still highly expressed in the TECs of the recipient thymus 10 months after a single dose of injection, although those in DCs could hardly be detected at this time point (Fig. 5f, g). Together, these data suggest that DMTV is efficient in removing donor-specific T cells even in the immune-competent recipient. The DMTV strategy therefore proves to be an efficient and feasible way to mitigate immune rejection of allogeneic organs or tissues, which has the potential to be applied in various clinical scenarios.

### DMTV mitigates immune rejection of allogeneic mouse embryonic stem cell transplants

**Fig. 6:**
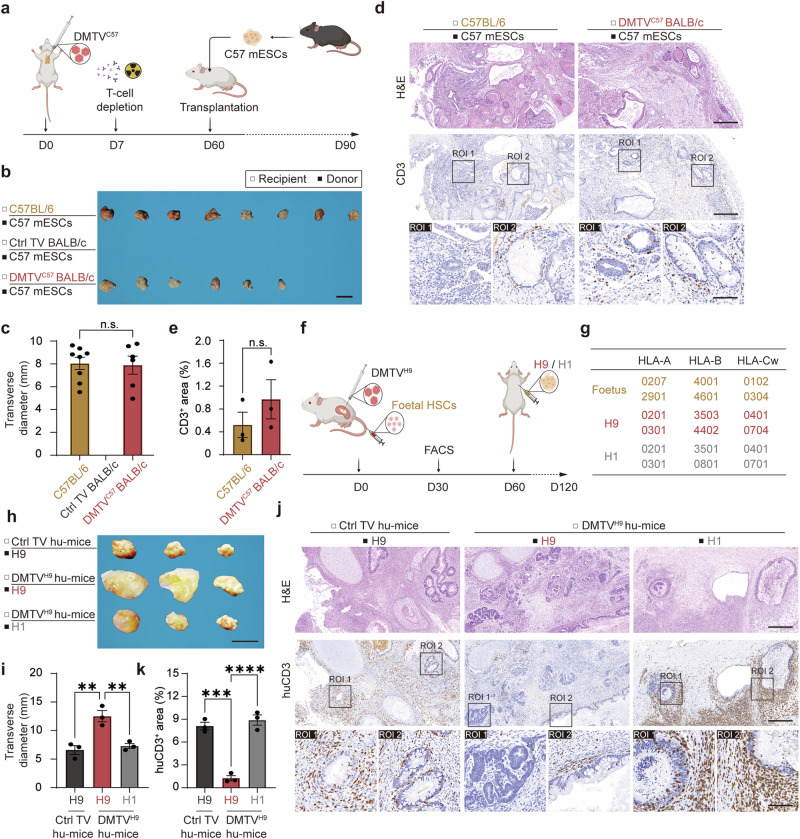
Donor-specific tolerance of allotransplanted multi-lineages mediated by DMTV. **a** Schematic representation of mESC transplantation. Day 0, thymus vaccination of Ctrl or C57BL/6 MHC; day 7, T cell depletion via anti-T cell antibodies and irradiation; day 60, mESC transplantation. Recipient, BALB/c mice receiving Ctrl TV or DMTV^C57^, or C57BL/6 mice; donor, C57BL/6 mESC. **b** Spontaneously developed teratomas generated from C57BL/6 mESC grafts in C57BL/6 mice and DMTV^C57^-treated BALB/c mice. No teratomas were generated in C57BL/6 mESC grafts in Ctrl TV-treated BALB/c mice. Scale bar, 1 cm. **c** Quantification of the transverse diameters of teratomas in **b**. Data are mean ± SEM (*n* = 8 and 6 in C57BL/6 mESC grafts from C57BL/6 mice and DMTV^C57^-treated BALB/c mice, respectively). Statistical significance was determined using two-tailed unpaired Student’s *t*-test. n.s., non-significant. **d** Representative H&E and IHC staining of CD3^+^ T cells in teratomas generated from C57BL/6 mESC grafts in C57BL/6 mice and DMTV^C57^-treated BALB/c mice. ROI 1, neural tube-like ectodermal tissues; ROI 2, intestine-like endodermal tissues. Scale bars of the top two panels, 400 μm; scale bar of the lower panel, 100 μm. **e** Quantification analyses of CD3-positively stained area in **d**. Data are mean ± SEM (*n* = 3 independent experiments). Statistical significance was determined using two-tailed unpaired Student’s *t*-test. n.s., non-significant. **f** Schematic representation of hESCs transplantation in a BLT humanized mouse model with either Ctrl TV or DMTV^H9^ in the reconstructed human thymus. Day 0, the M-NSG mice were transplanted with fetal thymus and liver tissues under the renal capsules and were tail vein injected with HSCs isolated from the same fetal liver. Before transplantation, the fetal thymus tissues were vaccinated with either Ctrl or H9 hESC HLA; day 30, the proportions of reconstituted human immune cells in the peripheral blood of the BLT-humanized mouse were validated via FACS; day 60, the BLT-humanized mouse with either Ctrl TV or DMTV^H9^ were subcutaneously transplanted with H1 or H9 hESCs. Recipient, BLT-humanized mouse treated with either Ctrl TV or DMTV^H9^; donor, H1 or H9 hESCs. **g** HLA typing of the fetal tissues, H1 hESCs and H9 hESCs. **h** Spontaneously developed teratomas generated from H1 and H9 hESC grafts in BLT-humanized mice treated with either Ctrl TV or DMTV^H9^. Scale bar, 1 cm. **i** Quantification of the transverse diameters of teratomas in **h**. Data are mean ± SEM (*n* = 3 for each group). Statistical significance was determined using the one-way ANOVA followed by Dunnett’s comparisons test. **P < 0.01; n.s., non-significant. **j** Representative H&E and IHC staining of huCD3^+^ T cells in teratomas generated from H1 and H9 hESC grafts in BLT-humanized mice treated with either Ctrl TV or DMTV^H9^. ROI 1, neural tube-like ectodermal tissues; ROI 2, intestine-like endodermal tissues. Scale bars of the top two panels, 400 μm; scale bar of the lower panel, 100 μm. **k** Quantification analyses of huCD3-positively stained area in **j**. Data are mean ± SEM (*n* = 3 independent experiments). Statistical significance was determined using the one-way ANOVA followed by Dunnett’s comparisons test. ****P < 0.0001; ***P < 0.001.

Owing to their pluripotency, mouse embryonic stem cells (mESCs) develop into almost all types of tissues or lineages after transplantation into immune-compromised mice. To investigate whether DMTV supports successful allogeneic transplantation of different tissues, we subcutaneously transplanted C57BL/6 mESCs into C57BL/6 mice (autologous transplantation) and Ctrl TV or DMTV^C57^ BALB/c mice (allogeneic transplantation) (Fig. 6a). In the autologous transplantation group, mESCs efficiently survived and developed into different lineages, such as ectodermal cells (region of interest 1, ROI 1) and endodermal cells (ROI 2) (Fig. 6b–e). In Ctrl TV BALB/c mice, allotransplanted C57BL/6 mESCs were completely rejected, which resulted in no transplants for further analyses. However, allotransplanted C57BL/6 mESCs in DMTV^C57^ BALB/c mice largely survived and normally developed into multi-lineages (Fig. 6b–e). Histological analyses further confirmed low T cell infiltration in both autologous transplants and allogeneic transplants in DMTV mice (Fig. 6d, e; Supplementary information, Fig. S6a, b). These results suggest that the donor-specific immune tolerance environment introduced by DMTV supports survival and normal development of mESCs. Meanwhile, DMTV-induced allotransplantation tolerance is universal and might be suitable for transplantation of various organs or tissues.

### Humanized DMTV also induces allotransplantation immune tolerance

To validate whether the DMTV strategy also functions in a human context, we vaccinated human donor MHC (referred to HLA hereafter) in the reconstructed human thymus of a BLT-humanized mouse model.^46,47^ NOD-Prkdc^scid^Il2rg^em1^/Smoc (M-NSG) mice were exposed to 1 Gy irradiation and transplanted with fetal thymus and liver tissues under the renal capsules. The thymus/liver-transplanted M-NSG mice were then tail vein injected with 5.0 × 10^5^ huCD34^+^ hemopoietic stem cells (HSCs) isolated from the liver of the same fetal donor (Fig. 6f). HLA typing revealed that HLA alleles of the fetal tissues used for constructing BLT-humanized mice were completely mismatched from both H1 and H9 human embryonic stem cells (WA01 and WA09 hESCs, authorized from WiCell, Madison) (Fig. 6g). Expression vectors for H9 HLA types were then constructed (AAV2/8-CMV-HLA-A*02:01-IRES-HLA-A*03:01, AAV2/8-CMV-HLA-B*35:03-IRES-HLA-B*44:02 and AAV2/8-CMV-HLA-Cw*04:01-IRES-HLA-Cw*07:04) (Supplementary information, Fig. S1).

To construct a humanized DMTV system, 1 × 10^11^ vg in 10 μL of each virus were co-injected into a block of fetal thymus (~100 mg) and the thymus was further incubated in 1 mL basal medium containing 2 × 10^11^ vg viruses for 2 h before renal subcapsular transplantation together with the liver tissue. FACS analysis revealed that human immune cells (huCD45^+^ and huCD3^+^) efficiently populated the peripheral blood of the humanized mice 2 months after H9 HLA DMTV (DMTV^H9^) and BLT humanized mouse model construction (Supplementary information, Fig. S6c, d). H1 or H9 hESCs were then subcutaneously injected into Ctrl TV or DMTV^H9^ BLT humanized mice. 2 months after hESC transplantation, the sizes of transplants recovered from DMTV^H9^ BLT humanized mice allotransplanted with H9 hESCs were obviously larger than those from DMTV^H9^ BLT humanized mice allotransplanted with H1 hESCs or Ctrl TV BLT humanized mice transplanted with H9 hESCs (Fig. 6h, i). Of note, there were prominent infiltration of huCD3^+^ T cells, huCD4^+^ T cells and huCD8^+^ T cells in ectodermal tissues (ROI 1) and endodermal tissues (ROI 2) of H1 hESC transplants from DMTV^H9^ BLT humanized mice and H9 transplants from Ctrl TV BLT humanized mice, whereas T cell infiltration could rarely be detected in tissues from H9 hESC transplants after DMTV^H9^ pre-treatment (Fig. 6j, k; Supplementary information, Fig. S6e, f). These data highlight that the DMTV strategy also functions in human context and could therefore serve as a potential strategy to bypass MHC-matching in organ allotransplantation.

## Discussion

**Fig. 7:**
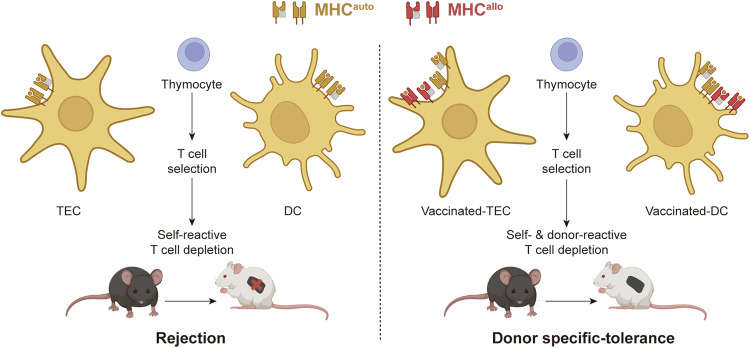
Donor MHC-specific thymus vaccination-induced immune tolerance for allogeneic transplantation. Under physiological conditions, the interactions between the TCRs on thymocytes and autologous MHC (MHC^auto^) on both TECs and DCs drive clonal deletion of self-reactive thymocytes and self-tolerance in mature T cells via positive and negative selection. MHC-mismatched allogeneic organ transplantation results in immune rejection due to the presence of donor-reactive T cells. The ectopic expression of donor allogeneic MHC (MHC^allo^) in TECs and/or DCs in the recipient thymus will recapitulate the selection process, which leads to specific depletion of donor-reactive T cells and promises immune tolerance in an allogeneic transplant.

Organ transplantation is well-acknowledged as the last-resort option to treat organ failure. However, the organ transplantation rate in patients left with this only option was extremely low owing to a lack of efficient system in finding and locating MHC-matched donors and subsequent difficulties in allocating, storing and transporting of recovered organs, which also resulted in severe underutilization of donated organs. Meanwhile, transplantation of partially MHC-matched organs in most cases leads to allograft rejection, which requires lifelong immunosuppressive treatment, shortening the survival period of transplanted organs and causing unwanted risks of infections and malignancies. One can therefore reasonably foresee that organ transplantation will ultimately become a regular treatment for patients with organ failure if the availability of donor organs is no longer an issue and the dilemma of immune rejection is overcome. In the current study, we performed ectopic expression of donor-specific MHC molecules in the recipient thymus to deplete donor-reactive T cells, which we referred to as the DMTV strategy. The strategy can achieve a smooth allotransplant without concern for immune rejection. Both in vitro and in vivo studies reveal that after DMTV, the recipient immune system becomes tolerant to donor organs or tissues although they bear a mismatched MHC profile. The DMTV strategy works together with the endogenous selection system in the thymus and educates T cells to tolerate both self MHC and donor MHC (Fig. 7). The DMTV strategy would therefore greatly simplify organ allotransplantation by avoiding stringent recipient-donor MHC matching and all other tedious procedures after recovering an organ, filling the gap standing between the recipient and the donor either spatially or temporally.

In a non-immune tolerant allotransplantation, T cells are activated directly or indirectly, which leads to activation of other immune cells and causes immune rejection.^48,49^ Removing donor-reactive T cells is therefore the key to ensure a successful allotransplantation. In the current study, DMTV efficiently removes donor-reactive T cells during their development in the thymus. This is evidenced by in vitro MLR analysis, in vivo DSA monitoring, and very low infiltration of T cells in the grafted tissues after DMTV even without any immunosuppressive intervention. Integrated scRNA-seq & scTCR-seq analysis further reveals clonal depletion of T cells harboring potential donor-reactive TCRs. Notably, the DMTV strategy only introduces immune tolerance to donor-specific MHC and animals receiving DMTV treatment show almost complete immune responses to general antigens as well as other allogeneic MHC variants. Using scRNA-seq & scTCR-seq, we also showed that in the peripheral T cell repertoire, the composition of T cell populations remained intact. Together, these data indicate that DMTV treatment has minor effects on overall T cell development and maturation. In the future, it will be intriguing to test whether the thymus vaccination strategy is also efficient to induce designed immune tolerance by expression of targeted antigens other than donor MHC in the thymus.

In humanized mice receiving DMTV treatment, cognate hESCs tolerate allogeneic transplantation and normally develop into multi-lineages. Although individualized human induced pluripotent stem cells (iPSCs) have been proposed for immunotolerant transplantation, it is expensive and needs long-period reprogramming procedures and safety validations. Engineering surface HLA molecule profiles or other immune modulatory molecules in human pluripotent stem cells (hPSCs) has also been proposed to generate universal cells for allogeneic transplantation.^50–53^ However, these engineered cells might be endowed with the capability of immune surveillance evasion. The DMTV strategy is another practical option of application of hESCs for cell therapies with no need of extra genetic engineering in these cells or immunosuppressive intervention. Thus, the DMTV strategy might be suitable not only for allogeneic organ transplantation but also for cell-based therapy utilizing products derived from hPSCs.

One of the major concerns of AAV-mediated gene therapy is AAV infection-induced production of anti-AAV neutralizing antibodies.^54^ In future studies, it is important to test whether long-term donor-specific immune tolerance would be achieved by one-dose of DMTV and whether repeated thymus AAV injection will lead to production of anti-AAV neutralizing antibodies. Before moving to clinical studies, it is equally important to validate the safety and efficacy of DMTV for organ allotransplantation in large animal models, such as non-human primates, with or without T cell depletion and the usage of immunosuppressive agents.

Banking of AAVs for all HLA-I variants expression is expected to save waiting time for efficient vaccination and transplantation. According to the European Molecular Biology Laboratory’s European Bioinformatics Institute (EMBL-EBI), 15,335 types of protein variants of HLA-I have so far been officially recognized in all populations.^55^ Considering future allogeneic transplantation between recipients and donors locally, the required expression vectors for various HLA proteins will be much reduced in a specific area. It is therefore doable for banking of all spectrums of AAVs in advance for each HLA protein expression, which will no doubt save waiting time for organ transplantation. Moreover, recent technological progresses have significantly shortened the time needed for HLA-typing to several hours.^56,57^ Even HLA-typing among deceased donors can also be done in shorter time so DMTV could be performed timely in recipients by immediate thymic injection of matched AAV vectors in the bank.

In summary, in the current study, we designed a DMTV strategy for constructing a donor MHC-specific immune tolerant system in recipients. The strategy could serve as a potent avenue to make maximum utilization of donated organs, increasing the immunocompatibility between the donor organs/cells and the recipient immune system before, during or after an allotransplantation, and minimizing the usage of immunosuppressants in the current allotransplantation paradigm.

## Materials and methods

### Animals

Female C57BL/6 (SM-001), BALB/c (SM-003), C3H/He (SM-008) and NOD-Prkdc^scid^Il2rg^em1^/Smoc (M-NSG) mice were purchased from Shanghai Model Organisms Center Inc, China. All mice were housed in groups of 5 individuals per cage and maintained on a 12-h light-dark cycle at 22–25 °C under specific-pathogen free (SPF) conditions. All animal experiments were approved by the Laboratory Animal Research Center, Tongji University. All procedures involving animals were carried out in compliance with the Guide for the Care and Use of Laboratory Animals, and ethical approval was granted by the Ethics Committee, Tongji University. BALB/c mice were thymus injected with AAVs at 7 weeks of age and received T cell depletion at 8 weeks of age. Skin tissue transplantation or pluripotent stem cell transplantation was performed at 4 months of age, 2 months after T cell depletion when the entire T cell repertoire has been re-constituted. The investigators were blinded to allocations during experiments and outcome assessment.

### Thymus vaccination

For donor MHC expression in recipient thymus, AAV packaging system was used. In brief, donor MHC cassettes driven by the CMV promoter were constructed in the AAV2/8 vector (Supplementary information, Fig. S1). After packaging, viruses were concentrated through gradient centrifugation and viral titer was detected by qRT-PCR (for rAAV genome).

Mice were anesthetized through intraperitoneal (i.p.) injection of Avertin. Hair on the chest was removed with depilatory cream. Mice were then intubated and connected to a small animal ventilator (RWD, cat. no. R420). After skin disinfection with povidone-iodine, a central skin incision at the level of 2^nd^ intercostal space was made. To expose the thymus, a horizontal incision at the mouse sternum was introduced and set apart with a retractor. AAVs for Ctrl TV or DMTV (1 × 10^13^ vg/mL, 10 μL for each MHC expression virus, and the same total dosage was applied for Ctrl in each group) were intrathymically injected with a 30-gauge Hamilton syringe. During the thymus vaccination procedure, mice were maintained inflated with a ventilator before thoracic cavity was closed and the opening was closely sutured. Carprofen (5 mg/kg, subcutaneous injection) and enrofloxacin (10 mg/kg, i.p. injection) were used to provide analgesia or prevent infection for 3 days after surgery.

### T cell depletion

1.5 mg anti-CD4 (BioXCell, New Hampshire, USA, BP0003-1) and 0.8 mg anti-CD8 (BioXCell, BP0061) monoclonal antibodies (mAbs) were i.p. injected into Ctrl TV mice or DMTV mice twice (7 days and 10 days after thymus vaccination) to deplete pre-existing CD4^+^ and CD8^+^ T cells, respectively. On day 10 after thymus vaccination, mice were subjected to a 3 Gy TBI. The populations of CD4^+^ and CD8^+^ T cells were then assessed by flow cytometry analysis.

### TEC, DC and PBMC isolation

To validate ectopic MHC expression in TECs and DCs, vaccinated thymus was isolated and cut into small pieces with scissors. Thymus tissues were then digested with 0.5 mg/mL papain (Sangon, Shanghai, China, cat. no. A003124), 2.5 mg/mL collagenase IV (R&D, cat. no. 9001-12-1) and 0.1 mg/mL DNase I (Sigma-Aldrich, Darmstadt, Germany, cat. no. 11284932001) in basal DMEM/F12 medium at 37 °C for 30 min. 10% fetal bovine serum (FBS) in DMEM/F12 was applied to stop the digestion and cells were passed through a 70-μm cell strainer before flow cytometry analysis.

PBMCs were collected for MLR assay, DSA detection, single-cell sequencing and immune cell composition detection. Peripheral blood cells and spleen homogenates (passed through a 40-μm cell strainer) were collected in tubes prefilled with EDTA. Histopaque®-1083 (Sigma-Aldrich, cat. no. 10831-100 mL) and Histopaque®-1077 (Sigma-Aldrich, cat. no. 10771-100 mL) were then used to enrich mouse and human PBMCs via density gradient centrifugation, respectively.

### Flow cytometry analysis

Flow cytometry analyses were performed as previously described. Cells were washed with flow cytometry buffer (PBS containing 2% BSA and 2 mM EDTA) and collected by centrifugation at 400× *g* for 5 min. Cells resuspended in flow cytometry buffer were then incubated with anti-mouse CD16/CD32 mAb (BD, New Jersey, USA, 553142) for 10 min on ice to block nonspecific FcR binding, followed by incubation of fluorescently-labeled antibodies for 30 min on ice. Flow cytometry was performed on a FACSVerse™ flow cytometer (BD). FlowJo Software was used for data analysis.

Antibodies used in flow cytometry analyses are as follows, CD45 (eBioscience, California, USA, cat. no. 12-0451-82; isotype: rat IgG2b kappa), CD45 (eBioscience, cat. no. 17-0451; isotype: rat IgG2b kappa), CD326 (BD, cat. no. 563478; isotype: rat IgG2a κ), CD11c (eBioscience, cat. no. 12-0114-81; isotype: Armenian hamster IgG), H2-Kb (eBioscience, cat. no. 11-5958-82; isotype: mouse IgG2a kappa), H2-Db (BD, cat. no. 553573; isotype: mouse IgG2b, κ), IA-b (Biolegend, San Diego, CA, cat. no. 116405; isotype: mouse IgG2a, κ), H2-Kd/Dd (Biolegend, cat. no. 34-1-2S; isotype: mouse IgG2a), CD3 (eBioscience, cat. no. 11-0031-63; isotype: Armenian hamster IgG), CD4 (BD, cat. no. 553051; isotype: rat IgG2a κ), CD8 (eBioscience, cat. no. 12-0081-82; isotype: rat IgG2a, κ), huCD45 (BD, cat. no. 555485; isotype: mouse IgG1, κ) and huCD3 (eBioscience, cat. no. 11-0038-42; isotype: mouse IgG1, κ).

### MLR assay

Isolated PBMCs were collected in X-VIVO™ 15 medium (Lonza, Visp, Switzerland, cat. no. 04-418Q) supplemented with 10% FBS and 200 U/mL pen/strep (Gibco, Massachusetts, USA, cat. no. 15140122). 1.0 × 10^5^ PBMCs from either Ctrl TV BALB/c or DMTV^C57^ BALB/c mice were labeled with CellTrace™ CFSE (Thermo Fisher Scientific, Massachusetts, USA, cat. no. C34554) and incubated with irradiated (25 Gy) 1.0 × 10^5^ PBMCs from BALB/c, C57BL/6 or C3H/He mice for 10 days in a well of 96-well flat-bottom culture plate in culture medium (X-VIVO™ 15, 10% FBS, 2mM _L_-glutamine (Gibco, cat. no. A2916801), 50 μM β-mercaptoethanol (Sigma-Aldrich, cat. no. 60-24-2), 20 U/mL IL-2 (Biolegend, cat. no. 575402) and 200 U/mL pen/strep. Phytohemagglutinin-L (PHA-L) (Invitrogen, Massachusetts, USA, cat. no. 00-4977-93) was served as a positive control. CFSE intensity in CD3^+^ T cells was checked on a FACSVerse flow cytometer (BD) and data were processed with FlowJo.

### Integrated scRNA-seq & scTCR-seq

10× Genomics platform was used for integrated scRNA-seq & scTCR-seq according to the manufacturer’s protocols. Libraries were sequenced by Illumina sequencer (Illumina, San Diego, CA) on a 150 bp paired-end run.

Cellranger (v7.1.0) was used to align reads to mm10 genome and generate feature-barcode matrices. Genes expressed in fewer than 3 cells were filtered from expression matrices. Cells with a mitochondrial fraction not in the highest confidence interval were filtered out, which results in removal of cells with a mitochondrial percentage of more than 5%.

Doublets were excluded with DoubletFinder (v2.0.3). Artificial doublets were generated from raw RNA count matrices based on the average of gene expression profiles of randomly sampled cell pairs. After merging artificial doublets with real existing scRNA-seq data using Seurat (v4.0.4), euclidean distance matrix was obtained from cell embeddings in principal component (PC) spaces. The proportion of artificial nearest neighbors (pANN) is computed by dividing its number of artificial neighbors by the neighborhood size (pK). Cells with the highest pANN were identified as doublets. The parameters were set as follows, where doublets proportion pN = 0.25, PCs = 30, pK was determined using mean-variance-normalized bimodality coefficient.

Seurat package (v4.0.4) was used for clustering. Raw RNA count matrices were normalized using SCTransform function with mitochondrial fraction as a variable to regress out. Top 2000 features that are repeatedly variable across datasets for integration were identified with SelectIntegration function. Anchors were then determined using the FindIntegrationAnchors() function and datasets were integrated together with IntegrateData() function. Dimensionality reduction was performed on the integrated data with principal component analysis (PCA). First 20 PCs were then used further for UMAP visualization and clustering procedure. The resolution of 0.1 was used in FindClusters function after computing the nearest neighbors by FindNeighbors function. Differentially expressed genes (DEGs) between clusters were identified using FindAllMarkers function. Cell type annotation was carried out with the expression of canonical gene markers.

Single-cell VDJ receptor sequences were assembled and analyzed with Cell Ranger’s vdj pipeline (v7.1.0). T cells with inappropriate combinations of α- and β-chains were removed. The expression levels of specific TCRs were assigned to cell populations defined with scRNA-seq clustering and visualized with UMAP.

### Monitoring DSAs

Monitoring DSAs was used for analyzing the overall immunoreactivity in DMTV-treated mice upon donor cell priming. Irradiated PBMCs from BALB/c, C57BL/6 and C3H/He mice were tail vein injected into the Ctrl TV BALB/c mice and DMTV^C57^ BALB/c mice. 10 days after inoculation, their sera were collected and incubated with the corresponding irradiated PBMCs at 37 °C overnight. FITC-anti-mouse secondary antibody (JacksonImmuno, Pennsylvania, USA, cat. no. 115-095-003) incubation was performed on the next day for 1 h at room temperature followed by flow cytometry analysis.

### Skin transplantation

For skin transplantation, mice were anaesthetized with 4% isoflurane (RWD, Shenzhen, China, cat. no. R510-22-10) in medical air and maintained under anesthesia using a nose cone with 1.5% isoflurane. Animals were placed on a heat pad set at 37 °C and hair was trimmed from the back of both recipient and donor. A 9 mm × 9 mm piece of full thickness skin was then cut off from the recipient back, and a 10 mm × 10 mm full thickness skin collected from the back of a donor was laid smoothly to align with the edge of the cut skin and was subsequently sutured together. Recipients after transplantation were patched with 3M nexcare (3M, Kleinostheim, Germany, cat. no. CBGBLRUS1509) and 3M athletic wrap (3M, cat. no. CBGBLRUS1507) was used for secondary fixation to make the skin better fit into the recipient graft bed. Carprofen and enrofloxacin were used to provide analgesia or prevent infection.

### Staining

Skin grafts and surrounding tissues or recovered teratomas were collected and fixed in 4% paraformaldehyde (PFA) at 4 °C overnight followed by gradient sucrose (in PBS) treatment. Tissues were then embedded in OCT compound (Sakura, California, USA, cat. no. 4583) and sectioned at 10-μm thickness using LEICA CM3050 S. For immunofluorescence staining, slides were incubated in blocking buffer (10% donkey serum, 0.1% Triton X-100 in PBS) for 1 h and incubated with primary antibodies at 4 °C overnight. After adequate washing with PBS, slides were incubated with fluorescently-conjugated secondary antibodies for 1 h at room temperature. Nuclei were counterstained with Hoechst 33258 (Sigma-Aldrich, D9542). Slides were then mounted with Fluoromount-G Mounting Medium (Southern Biotech, Alabama, USA, cat. no. 0100-01). Images were captured using confocal microscope (Leica SP8).

For hematoxylin and eosin (H&E) staining, tissues were fixed in 4% PFA and subjected to paraffin embedding. Paraffin-embedded tissues were sectioned at 3-μm thickness, and sections were processed for H&E staining.

The following primary antibodies were used for staining analyses in the current study, anti-CD3 (Servicebio, Wuhan, China, GB13014), anti-CD4 (Servicebio, GB13064-2) and anti-CD8 (Servicebio, GB114196).

### Construction of humanized mice

BLT-humanized mouse model was constructed according to a previously published protocol.^46^ Normal aborted fetuses were obtained from Shanghai First Maternity and Infant Hospital or Jing’an District Hospital of Traditional Chinese Medicine with agreement of the donors and approval of related ethical review and informed consent documents. All the procedures were approved by the Ethics Committee of School of Medicine, Tongji University, and complied with the fundamental guidelines for the proper conduct of Interim Measures for the Administration of Human Genetic Resources and related activities in academic research institutions under the jurisdiction of the Chinese Ministry of Health.^58^ HLA typing (HLA-A, -B and -Cw) of human fetus was performed by the Shanghai Tissuebank Biotechnology Co., Ltd. M-NSG mice were exposed to 1 Gy irradiation right before renal subcapsular transplantation of fetal liver and thymus tissues at the right side. The thymus/liver-transplanted M-NSG mice were then tail vein injected with 5.0 × 10^5^ huCD34^+^ HSCs enriched with magnetic beads (Meltenyi, Bergisch Gladbach, Germany, 130-056-701) from the liver of the same fetal donor.

### Teratoma formation

mESCs or hESCs were injected subcutaneously over the scapula in recipient mice as indicated at a dosage of 1.0 × 10^6^ cells for mESCs and 2.0 × 10^6^ cells for hESCs per injection site.

### Statistical analysis

Statistical analyses were carried out with GraphPad Prism 9. Differences among groups were evaluated by Student’s *t*-test, One-way ANOVA or Log-rank test as indicated in each figure legend. One-way ANOVA of variance with a Dunnett’s multiple comparisons test was performed to identify statistical significance among groups of samples. All data are presented as mean ± SEM. Significance was determined with ****P < 0.0001; ***P < 0.001; **P < 0.01; *P < 0.05.

## Supplementary information

Supplementary information, Fig. S1 AAV delivery system designed for thymus vaccination.

Supplementary information, Fig. S2 FACS analysis of the depletion and reconstitution of TCR repertoire.

Supplementary information, Fig. S3 Immunoreactive analysis of the reconstituted T cells after thymus vaccination.

Supplementary information, Fig. S4 DMTV ameliorates T cell infiltration in allotransplanted mouse skin.

Supplementary information, Fig. S5 DMTV-induced tolerance in sensitized recipients and recipients without T cell depletion in advance.

Supplementary information, Fig. S6 DMTV ameliorates T cell infiltration in allogeneic mouse and human embryonic stem cell-originated multi-lineages.

## Data Availability

The raw sequencing data reported in this manuscript have been deposited in the Genome Sequence Archive in National Genomics Data Center, China National Center for Bioinformation/Beijing Institute of Genomics, Chinese Academy of Sciences (GSA: CRA019607) that are publicly accessible at https://ngdc.cncb.ac.cn/gsa/.

## Code availability

The code used in this study is available from the corresponding author upon reasonable request.
