# Supplementary material for: Donor MHC-specific thymus vaccination allows for immunocompatible allotransplantation
Source: Cell Res. 2025 Jan 3;35(2):132–44. doi: 10.1038/s41422-024-01049-5 (PMC11770082; doi:10.1038/s41422-024-01049-5)
Supplement: Supplementary file 1 — Supplementary information, Fig. S1 AAV delivery system designed for thymus vaccination. [file 41422_2024_1049_MOESM1_ESM.pdf]

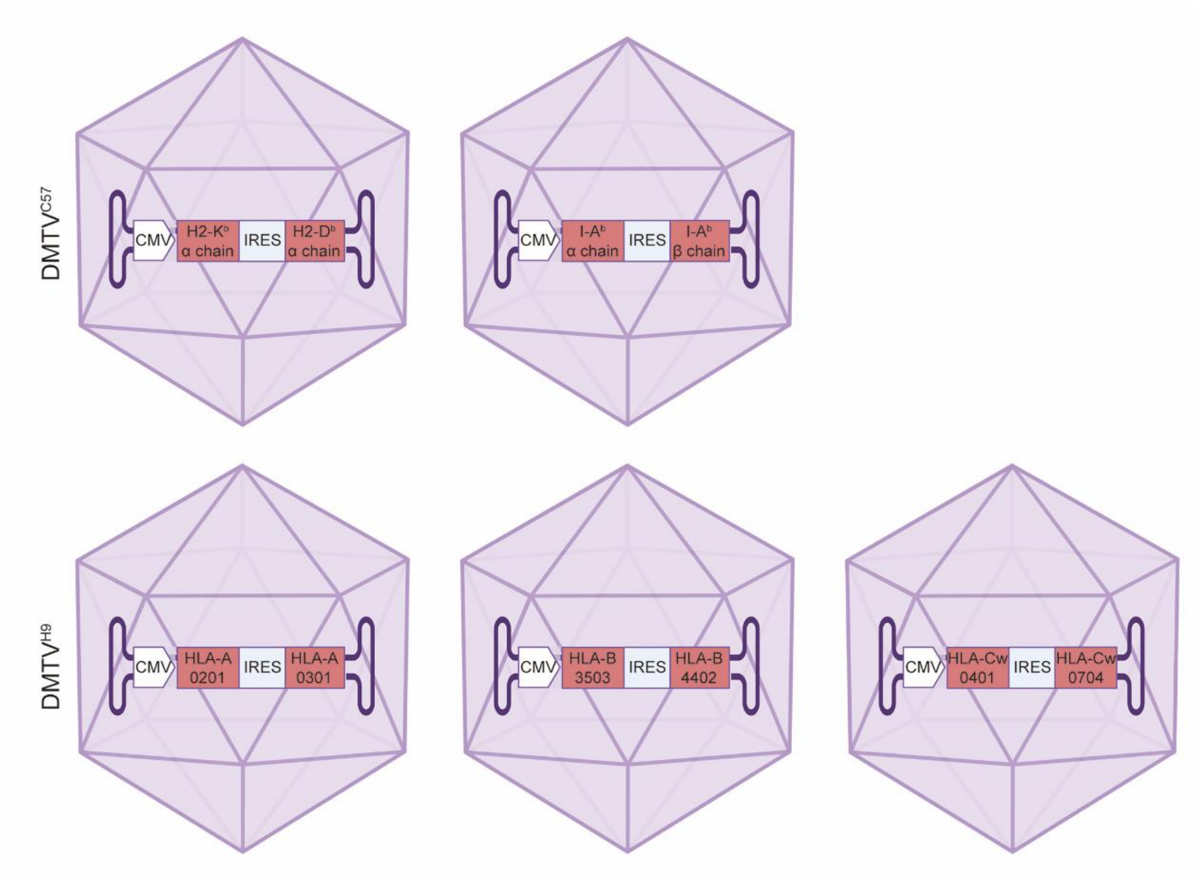

Fig. S1 AAV delivery system designed for thymus vaccination.

An AAV2/8-CMV-H2-K^b α chain-IRES-H2-D^b α chain was constructed to express MHC class I molecules of C57BL/6 mice. An AAV2/8-CMV-I-A^b α chain-IRES-I-A^b β chain was constructed to express MHC class II molecules of C57BL/6 mice. AAV2/8-CMV-HLA-A0201-IRES- HLA-A0301, AAV2/8-CMV-HLA-B3503-IRES- HLA-B4402, AAV2/8-CMV-HLA-Cw0401-IRES- HLA-Cw0704 were constructed to express MHC class I molecules of H9 hESCs.
