# Supplementary material for: Donor MHC-specific thymus vaccination allows for immunocompatible allotransplantation
Source: Cell Res. 2025 Jan 3;35(2):132–44. doi: 10.1038/s41422-024-01049-5 (PMC11770082; doi:10.1038/s41422-024-01049-5)
Supplement: Supplementary file 2 — Supplementary information, Fig. S2 FACS analysis of the depletion and reconstitution of TCR repertoire. [file 41422_2024_1049_MOESM2_ESM.pdf]

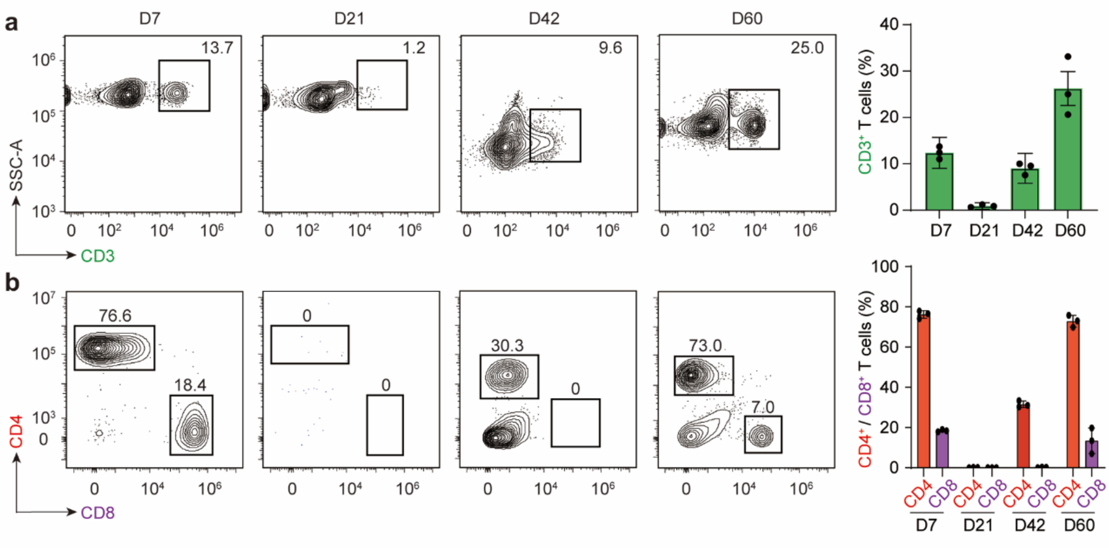

Fig. S2 FACS analysis of the depletion and reconstitution of TCR repertoire.

After thymus vaccination in recipient BALB/c mice, anti-CD4 and anti-CD8 mAbs were used to deplete T cells, and the proportions of $CD3^+$ T cells **(a)**, $CD4^+$ and $CD8^+$ T cells **(b)** in PBMCs were analyzed with FACS along the T cell reconstitution process. Representative flow cytometry plots illustrating T cell populations in peripheral PBMCs after T cell depletion (left panel); Quantification analyses of percentages of $CD3^+$, $CD4^+$ and $CD8^+$ T cells (right panel). Data are mean ± SEM (*n*=3 independent experiments).
