# Supplementary material for: Donor MHC-specific thymus vaccination allows for immunocompatible allotransplantation
Source: Cell Res. 2025 Jan 3;35(2):132–44. doi: 10.1038/s41422-024-01049-5 (PMC11770082; doi:10.1038/s41422-024-01049-5)
Supplement: Supplementary file 3 — Supplementary information, Fig. S3 Immunoreactive analysis of the reconstituted T cells after thymus vaccination. [file 41422_2024_1049_MOESM3_ESM.pdf]

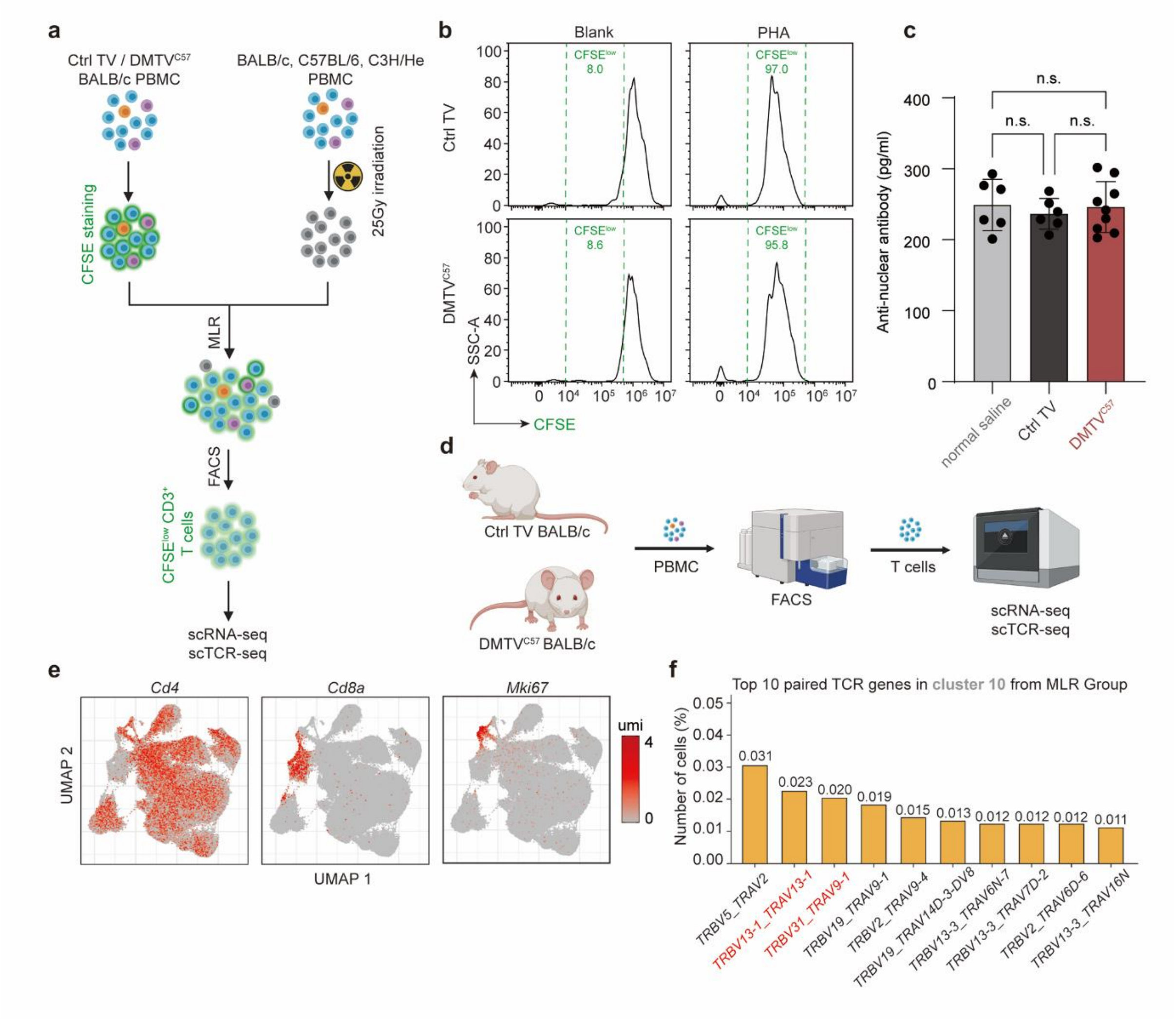

Fig. S3 Immunoreactive analysis of the reconstituted T cells after thymus vaccination.

a Schematic representation of mixed lymphocyte reaction (MLR) analysis followed by integrative single-cell RNA sequencing (scRNA-seq) and single-cell TCR sequencing (scTCR-seq) to capture clonally expanded T cells or TCRs. PBMCs from thymus vaccinated BALB/c mice were stained with the intracellular fluorescent label carboxyfluorescein diacetate succinimidyl ester (CFSE) and incubated with irradiated PBMCs from BALB/c, C57BL/6 or C3H/He mice for 10 days. $CD3^{+}$ $CFSE^{low}$ T cells were considered as the clonally expanded T cell populations, which were FACS enriched for integrated scRNA-seq & scTCR-seq.

b MLR analysis in $CD3^{+}$ T cells isolated from Ctrl TV- and $DMTV^{C57}$-treated BALB/c mice revealed comparable immunoreactivity to common antigen phytohemagglutinin (PHA).

c Serum anti-nuclear antibody (ANA) levels of BALB/c mice with normal saline thymus injection (n=6), Ctrl TV (n=6) and $DMTV^{C57}$ (n=9) for 2 months were measured by ELISA. Data are mean ± SEM. Statistical significance was determined using the one-way ANOVA followed by Dunnett's comparisons test. non-

significant (n.s.).

d Schematic representation of integrated scRNA-seq & scTCR-seq in $CD3^+$ T cells isolated from PBMCs of Ctrl TV- or $DMTV^{C57}$-treated BALB/c mice.

e FeaturePlot analyses showed *Cd4*, *Cd8a* and *Mki67* gene expression in T cell clusters in mixed lymphocyte reaction (MLR, n=2, 23 709 cells) and peripheral T cells from Ctrl TV (n=3, 83 485 cells) and $DMTV^{C57}$ BALB/c mice (n=3, 30 241 cells).

f TRAV_TRBV pairing profiles in clonally expanded Cluster #10 T cells in MLR. *TRBV13-1_TRAV13-1* and *TRBV31_TRAV9-1*, TCR pairs enriched in clonally expanded Cluster #9 $CD8^+$ T cells and Cluster #1 $CD4^+$ T cells after C57BL/6 PBMC priming, were also enriched in clonally expanded Cluster #10 proliferating T cells.
