# Supplementary material for: Donor MHC-specific thymus vaccination allows for immunocompatible allotransplantation
Source: Cell Res. 2025 Jan 3;35(2):132–44. doi: 10.1038/s41422-024-01049-5 (PMC11770082; doi:10.1038/s41422-024-01049-5)
Supplement: Supplementary file 4 — Supplementary information, Fig. S4 DMTV ameliorates T cell infiltration in allotransplanted mouse skin. [file 41422_2024_1049_MOESM4_ESM.pdf]

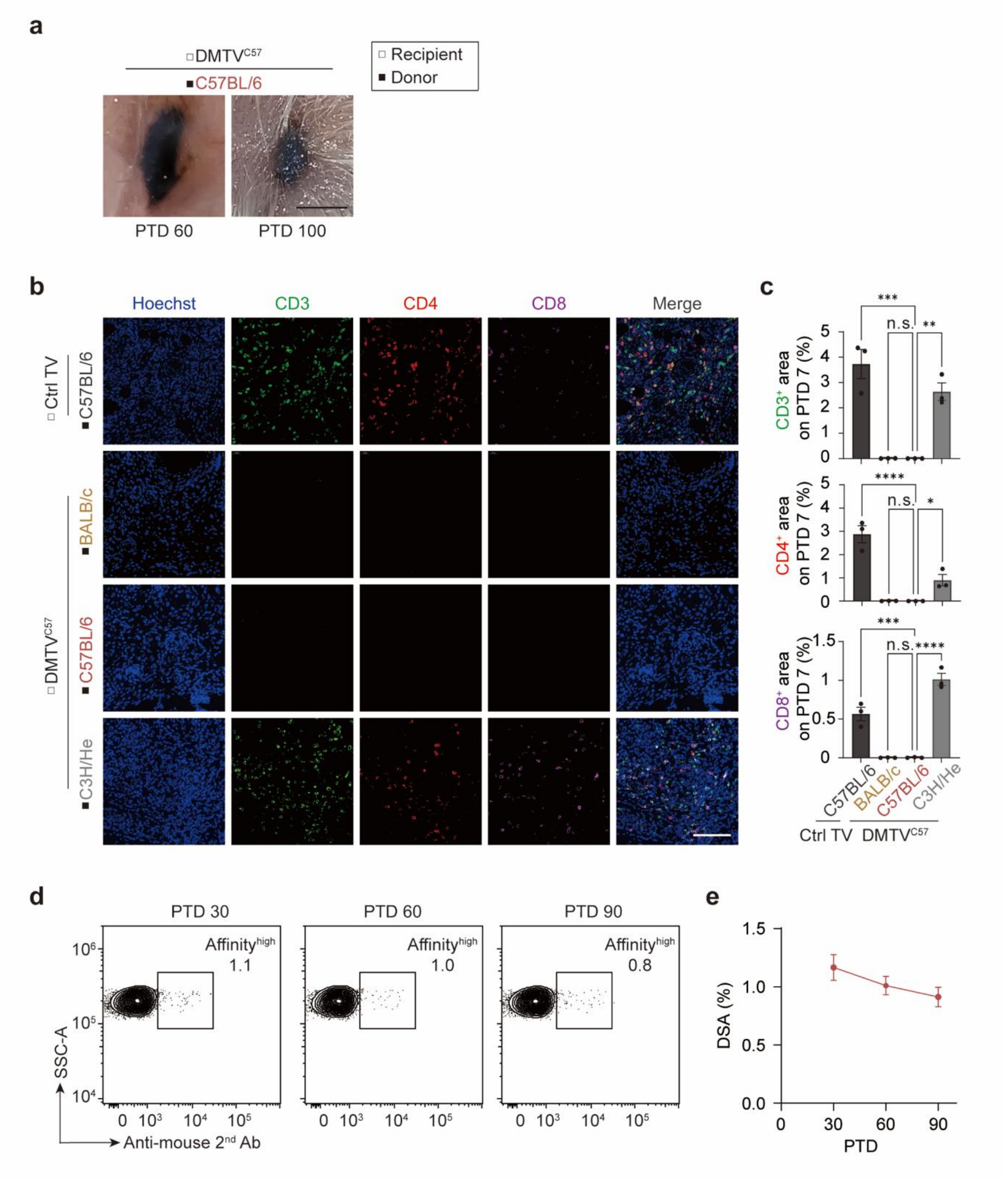

Fig. S4 DMTV ameliorates T cell infiltration in allotransplanted mouse skin.

a Images of skin grafts 60 and 100 days after transplantation. Scale bar, 5 mm.

b Representative immunofluorescence (IF) staining of $CD3^+$, $CD4^+$ and $CD8^+$ T cells in slices of skin tissues 7 days after transplantation. Scale bar, 100 μm.

c Quantification analyses of CD3, CD4 and CD8 positively stained area 7 days after transplantation in **a**. Data are mean ± SEM (*n*=3 independent experiments). Statistical significance was determined using the one-

way ANOVA followed by Dunnett's comparisons test. ****$P < 0.0001$; ***$P < 0.001$; **$P < 0.01$; *$P <$ 0.05; non-significant (n.s.).

d Representative flow cytometry plot of DSA detection, where Affinity^{high} indicates the presence of DSA in the serum.

e Quantification of proportions of cells with high DSA affinity. Data are mean ± SEM (*n*=3 independent experiments).
