# Supplementary material for: Donor MHC-specific thymus vaccination allows for immunocompatible allotransplantation
Source: Cell Res. 2025 Jan 3;35(2):132–44. doi: 10.1038/s41422-024-01049-5 (PMC11770082; doi:10.1038/s41422-024-01049-5)
Supplement: Supplementary file 5 — Supplementary information, Fig. S5 DMTV-induced tolerance in sensitized recipients and recipients without T cell depletion in advance. [file 41422_2024_1049_MOESM5_ESM.pdf]

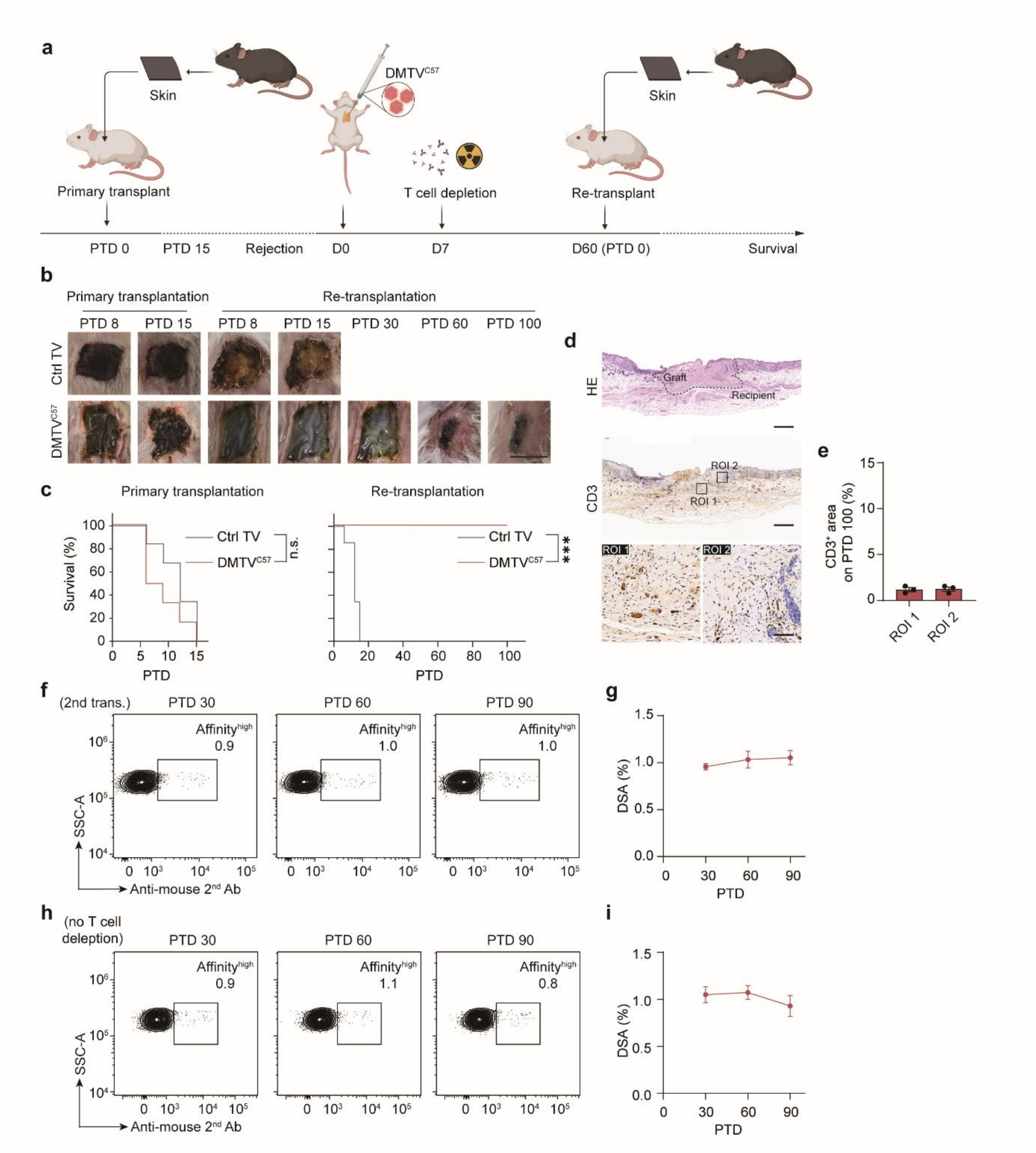

Fig. S5 DMTV-induced tolerance in sensitized recipients and recipients without T cell depletion in advance.

a Schematic representation of the primary transplantation to sensitize the recipient to donor antigens, DMTV, and secondary transplantation.

b Representative skin tissues 8, 15 days after primary transplantation and representative skin tissues 30, 60 and 100 days after secondary transplantation. Scale bar, 1cm.

c Survival curves of the donor skin grafts. Data are mean ± SEM (*n*=6 independent experiments). Primary

transplantation: non-significant (n.s.); Secondary transplantation: ***$P < 0.001$; Log-rank test.

d Representative H&E and IHC staining of $CD3^+$ T cells in adjacent slices from mice 100 days after transplantation. ROI 1, the region of the donor skin graft; ROI 2, the junction region between recipient skin and donor skin. Scale bars of the top two panels, 500 μm; scale bar of the lower panel, 40 μm.

e Quantification analyses of CD3 positively stained area 100 days after transplantation in ROI 1 and ROI 2 in **d**. Data are mean ± SEM (n=3 independent experiments).

f Representative flow cytometry plot of DSA detection at different time points after DMTV and skin tissue secondary transplantation, where $Affinity^{high}$ indicates the presence of DSAs in the serum.

g Quantification of proportions of cells with high DSA affinity in **f**. Data are mean ± SEM (n=3 independent experiments).

h Representative flow cytometry plot of DSA detection at different time points after DMTV and skin tissue transplantation without T cell depletion in advance, where $Affinity^{high}$ indicates the presence of DSAs in the serum.

i Quantification of proportions of cells with high DSA affinity in **h**. Data are mean ± SEM (n=3 independent experiments).
