# Supplementary material for: Donor MHC-specific thymus vaccination allows for immunocompatible allotransplantation
Source: Cell Res. 2025 Jan 3;35(2):132–44. doi: 10.1038/s41422-024-01049-5 (PMC11770082; doi:10.1038/s41422-024-01049-5)
Supplement: Supplementary file 6 — Supplementary information, Fig. S6 DMTV ameliorates T cell infiltration in allogeneic mouse and human embryonic stem cell-originated multi-lineages. [file 41422_2024_1049_MOESM6_ESM.pdf]

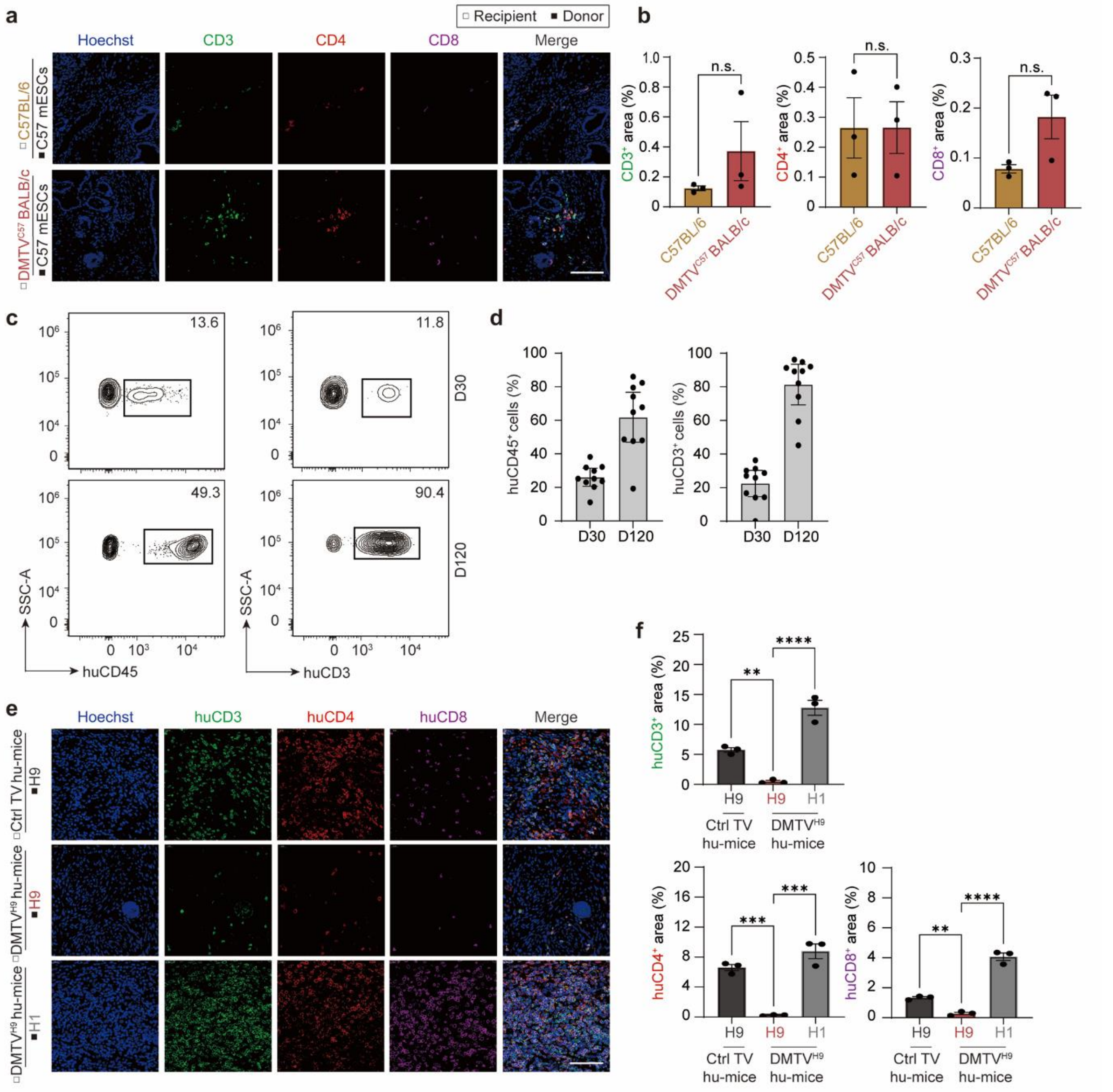

Fig. S6 DMTV ameliorates T cell infiltration in allogeneic mouse and human embryonic stem cell-originated multi-lineages.

a Representative IF staining of CD3$^+$, CD4$^+$, CD8$^+$ T cells in teratomas originated from C57BL/6 mouse embryonic stem cell (mESC) subcutaneous transplantation in C57BL/6 mice and DMTV^{C57}-treated BALB/c mice. Scale bar, 100 μm.

b Quantification analyses of proportions of infiltered CD3$^+$, CD4$^+$, CD8$^+$ T cells in teratomas in **a**. Data are mean ± SEM (*n*=3 independent experiments). Statistical significance was determined using two-tailed unpaired Student's *t* test. non-significant (n.s.).

c Representative FACS analysis of proportions of huCD45$^+$ cells in PBMCs and huCD3$^+$ cells in huCD45$^+$ cells in BLT-humanized mice at Day 30 and Day 120.

d Quantification analyses of $huCD45^+$ cells in PBMCs and $huCD3^+$ cells in $huCD45^+$ cells in **c**. Data are mean ± SEM (n=10 independent experiments).

e Representative IF staining of $huCD3^+$, $huCD4^+$, $huCD8^+$ T cells in H9 hESC-derived teratomas in the Ctrl TV-treated BLT-humanized mice or H9 and H1 hESC-derived teratomas in $DMTV^{H9}$-treated BLT-humanized mice. Scale bar 100μm.

f Quantification analyses of proportions of infiltered $huCD3^+$, $huCD4^+$, $huCD8^+$ T cells in teratomas in **e**. Data are presented as mean ± SEM; n=3 independent experiments. Statistical significance was determined using the one-way ANOVA followed by Dunnett's comparisons test. ****$P < 0.0001$; ***$P < 0.001$; **$P < 0.01$.
